# Systematic benchmark of state-of-the-art variant calling pipelines identifies major factors affecting accuracy of coding sequence variant discovery

**DOI:** 10.1186/s12864-022-08365-3

**Published:** 2022-02-22

**Authors:** Yury A. Barbitoff, Ruslan Abasov, Varvara E. Tvorogova, Andrey S. Glotov, Alexander V. Predeus

**Affiliations:** 1 Bioinformatics Institute, St. Petersburg, Russia; 2 Department of Genomic Medicine, D.O. Ott Research Institute of Obstetrics, Gynaecology and Reproductology, St. Petersburg, Russia; 3 Department of Genetics and Biotechnology, St. Petersburg State University, St. Petersburg, Russia; 4 Dmitry Rogachev National Research Center of Pediatric Hematology-Oncology and Immunology, Moscow, Russia

**Keywords:** Whole genome sequencing, Whole exome sequencing, Variant calling, Pipeline, Benchmark, Performance comparison, Genome in a Bottle

## Abstract

**Background:**

Accurate variant detection in the coding regions of the human genome is a key requirement for molecular diagnostics of Mendelian disorders. Efficiency of variant discovery from next-generation sequencing (NGS) data depends on multiple factors, including reproducible coverage biases of NGS methods and the performance of read alignment and variant calling software. Although variant caller benchmarks are published constantly, no previous publications have leveraged the full extent of available gold standard whole-genome (WGS) and whole-exome (WES) sequencing datasets.

**Results:**

In this work, we systematically evaluated the performance of 4 popular short read aligners (Bowtie2, BWA, Isaac, and Novoalign) and 9 novel and well-established variant calling and filtering methods (Clair3, DeepVariant, Octopus, GATK, FreeBayes, and Strelka2) using a set of 14 “gold standard” WES and WGS datasets available from Genome In A Bottle (GIAB) consortium. Additionally, we have indirectly evaluated each pipeline’s performance using a set of 6 non-GIAB samples of African and Russian ethnicity. In our benchmark, Bowtie2 performed significantly worse than other aligners, suggesting it should not be used for medical variant calling. When other aligners were considered, the accuracy of variant discovery mostly depended on the variant caller and not the read aligner. Among the tested variant callers, DeepVariant consistently showed the best performance and the highest robustness. Other actively developed tools, such as Clair3, Octopus, and Strelka2, also performed well, although their efficiency had greater dependence on the quality and type of the input data. We have also compared the consistency of variant calls in GIAB and non-GIAB samples. With few important caveats, best-performing tools have shown little evidence of overfitting.

**Conclusions:**

The results show surprisingly large differences in the performance of cutting-edge tools even in high confidence regions of the coding genome. This highlights the importance of regular benchmarking of quickly evolving tools and pipelines. We also discuss the need for a more diverse set of gold standard genomes that would include samples of African, Hispanic, or mixed ancestry. Additionally, there is also a need for better variant caller assessment in the repetitive regions of the coding genome.

**Supplementary Information:**

The online version contains supplementary material available at 10.1186/s12864-022-08365-3.

## Background

Over the past decade next-generation sequencing (NGS) has become a widely used technique in genetics and genomics [1]. Rapid technology development, as well as the introduction of whole-exome sequencing (WES) and target gene sequencing panels, facilitated NGS application to the analysis of human genome variation and molecular diagnostics of inherited disease. Millions of individual exomes and genomes have been sequenced across the globe, and large-scale variant datasets have been constructed from NGS data, including the Genome Aggregation Database (gnomAD) [2] and UK Biobank exome sequencing dataset [3]. These datasets are extensively used in both clinical practice and basic human genetics research.

Despite huge developments over the past years, accuracy and reliability of variant discovery (variant calling) from NGS data still has room for improvement. Any basic variant calling pipeline includes two key stages: read alignment against a reference genome sequence and variant calling itself. Hence, quality of the reference genome sequence [4] as well as properties of the software tools used for read alignment and variant calling all influence the final result. While BWA is considered a gold standard solution for short read alignment in medical genetics [5], several other aligners have been developed and are commonly used, including Bowtie2 [6], Isaac (Illumina Inc. USA), and Novoalign (Novocraft Technologies, USA). The spectrum of software tools for variant calling is much broader, ranging from relatively simple (such as the SAMtools/BCFtools pipeline [7]) to rather complex ones (e.g., Genome Analysis ToolKit (GATK) [5, 8, 9] HaplotypeCaller based on local haplotype assembly and Markov model-based genotyping). Active development of deep learning models and their application to biological data led to the introduction of neural network-based variant discovery methods such as DeepVariant [10]. Variant filtration methods based on convolutional neural networks are now also available in the most recent versions of GATK.

Existence of multiple variant calling pipelines predicates the need for a gold standard genome variation dataset that can be used for extensive benchmarking of variant discovery pipelines. Such a gold standard dataset has been compiled by the Genome In A Bottle Consortium (GIAB) and the National Institute of Standards (NIST) [11]. The dataset includes high-confidence genotypes for a set of samples (the European NA12878/NA12891/NA12892 trio, the Chinese trio, and the Ashkenazi trio) obtained using multiple genotyping strategies. These high-confidence variant calls can be used as a truth set to evaluate the accuracy of variant calling, and estimate the precision and sensitivity of variant discovery. The GIAB gold standard dataset has been used multiple times for benchmarking of variant detection solutions. For example, a 2015 study by Hwang et al. [12] used a set of sequencing datasets of the NA12878 sample and demonstrated important differences in the accuracy of variant calling pipelines available at the time, with a combination of BWA-MEM and SAMtools being the best solution for SNP calling, and BWA-MEM and GATK-HC for indels. More recently, several comparative analyses have shown that DeepVariant and Strelka2 [13] show the best performance on individual GIAB samples [14–16]. The most recent comparative evaluation also demonstrated the utility of combining variant calling results from several pipelines [16]. While the aforementioned studies provide important information regarding the performance of different software, a single gold standard sample (NA12878) is usually used for comparison. This limitation does not allow estimation of the robustness of different pipelines and their ability to call variants in samples of different origin and/or sequencing quality.

In 2019, best practices for benchmarking variant calling software have been developed by the Genome Alliance for Genomics and Health (GA4GH) [17]. A reference implementation of the GA4GH benchmarking strategy, hap.py, allows researchers to evaluate the performance of a variant calling pipeline in a stratified manner, i.e. compare the accuracy of variant discovery in different sets of regions and for different variant types [17]. Such a stratified approach provides important information regarding the major factors affecting variant discovery. This, in turn, gives an opportunity to conduct a systematic survey of factors affecting reliable variant discovery. Previously, we have conducted a detailed analysis of the determinants of human coding sequence coverage in WES and WGS [18]. This study showed that all modern approaches to human genome resequencing have reproducible coverage bias, and mappability limitations are its major drivers. These results prompted us to investigate the influence of different sequence-based factors and coverage biases on the performance of variant calling software. To this end, in this work we applied 45 different combinations of read alignment, variant calling, and variant filtration tools to a set of 14 gold standard samples from the GIAB data (630 VCF files overall), and evaluated their robustness and general performance across different sets of human coding sequences.

## Results

### Data collection and analysis strategy

**Fig. 1:**
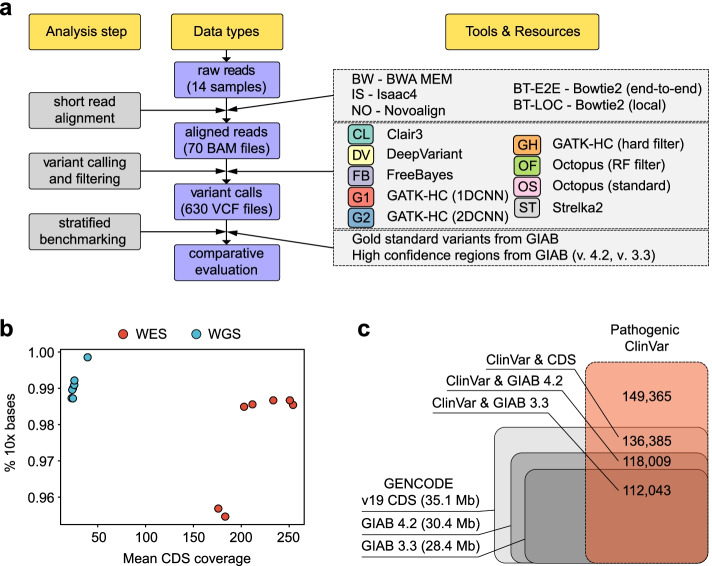
Systematic benchmarking of multiple variant calling pipelines. **a** A chart representing the analysis workflow. **b** A scatterplot showing mean coverage of high-confidence coding sequence regions (defined by the Genome In A Bottle consortium) and the fraction of bases of such regions covered at least 10x total read depth in WGS and WES datasets used (each point corresponds to an individual sample). **c** Reciprocal overlap of GENCODE v19 CDS intervals, GIAB v. 4.2 and GIAB v. 3.3 high confidence regions common for all 7 samples, and pathogenic/likely pathogenic variants without conflicting reports from ClinVar (ClinVar v. 20,211,130 was used)

To dissect the factors that define the accuracy of variant calling, we selected a matching set of WES and WGS datasets available for the gold standard GIAB samples, including NA12878 (HG001), three members of an Ashkenazi trio (HG002 - HG004), and three members of the Chinese Han trio (HG005 - HG007) (Fig. 1a, Table 1). All WES datasets used in the analysis were generated using similar capture kits (Agilent SureSelect All Exon v5 or v7), and all WGS samples were done using PCR-free WGS technology. While all considered exome samples had high (100-200x) coverage, we have restricted WGS coverage to more realistic 30-50x (Fig. 1b).

The following analysis strategy was employed for each sample (Fig. 1a): raw reads were aligned onto the GRCh37 human reference genome with either of the four short read aligners: Bowtie2 (in either end-to-end (BT-E2E) or local (BT-LOC) alignment mode), BWA MEM (BW), Isaac4 (IS), and Novoalign (NO). Resulting alignment files in BAM format were subject to pre-processing with GATK to mark duplicate reads, and were then processed with six different variant callers, including both well-established (DeepVariant (DV), Strelka2 (ST), GATK-HC (denoted as G1, G2, or GH depending on the filtering strategy), and FreeBayes (FB)), and more recently developed Clair3 (CL) and Octopus (OS and OF for standard filtering and random forest filtering, respectively) (see Methods for details). Raw variant calls were subject to filtering with standard built-in filters or quality-based filtering (see Methods), and filtered variant call sets were then evaluated using the hap.py toolkit, with an additional stratification of coding regions by expected read depth, GC content, mappability, and other factors (see below).

The benchmarking was performed on both the most up-to-date GIAB v4.2 high-confidence regions, as well as the older GIAB v3.3 which includes less challenging sequences. For uniformity of the analysis, high-confidence regions for individual samples were intersected to obtain a single set of regions to be used when evaluating all samples. Such a set of high-confidence regions included 30.4 Mbp of coding sequence for GIAB v4.2 and 28.4 Mbp - for GIAB v3.3 (Fig. 1c). The high-confidence regions cover more than 75% (for GIAB v3.3) or 79% (for GIAB v4.2) known pathogenic variants from the ClinVar database (Fig. 1c) and are thus most relevant to clinically significant variant discovery.

Prior to the analysis of benchmarking results, we compared the overall quality and coverage of coding sequences in WES and WGS samples used. As the WES dataset for HG001 contained more than 250 million reads, we randomly selected 40% of all read pairs prior to the analysis. All WES samples were characterized with significantly higher mean coverage of CDS regions and had slightly lower percentages of regions covered with at least 10 reads (Fig. 1b, Table 1), with the exception of HG006 and HG007 that were characterized with a narrower coverage (95.5 and 95.7% of high-confidence regions covered at 10x), possibly due to minor differences in the capture protocol for these samples. At the same time, the fraction of bases with at least 20x coverage was higher in WES than in WGS (Supplementary Fig. 1). Overall, these results suggest that the difference in the estimated variant calling performance on WES and WGS data should not be driven by general low coverage in WES and might be in part attributed to CDS regions not included into the WES capture kit design.

**Table 1:** Descriptive statistics of the gold standard sequencing datasets used in the study

Sample	Type	Source (SRA ID)	Mean coverage	Fraction of 10x bases	Median called variant count^b^	True variant count
HG001	WGS	GIAB FTP	22.2	0.987	20,422	20,444
	WES	ERR1905890	248.8^a^	0.985^a^	19,875	
HG002	WGS	GIAB FTP	23.2	0.990	20,651	20,647
	WES	SRR2962669	241.4	0.987	20,048	
HG003	WGS	GIAB FTP	23.2	0.987	20,623	20,660
	WES	SRR2962692	203.9	0.987	20,046	
HG004	WGS	GIAB FTP	22.8	0.990	20,729	20,745
	WES	SRR2962694	228.4	0.987	20,112	
HG005	WGS	GIAB FTP	37.3	0.998	20,650	20,620
	WES	SRR2962693	195.5	0.985	19,969	
HG006	WGS	GIAB FTP	25.5	0.991	20,320	20,354
	WES	SRR14724507	183.2	0.955	19,650	
HG007	WGS	GIAB FTP	25.6	0.992	20,483	20,526
	WES	SRR14724506	176	0.957	19,793	

Coverage and variant statistics are given with respect to GIAB v4.2 high-confidence CDS regions. ^a^Coverage values for downsampled HG001 WES dataset are given (see Methods); ^b^variant counts were obtained by calculating the median number of variants discovered by different pipelines. Full statistics for each sample and tool combination is given in Supplementary Table S1

We then evaluated the total number of variants discovered inside the high-confidence protein-coding regions with each of the 45 variant discovery pipelines used. All samples had a comparable number of variant calls, with a median value slightly above or below 20,000 variants per sample (Supplementary Table S1). In all cases, fewer variants were discovered for WES samples compared to WGS (Table 1). For most samples the difference in variant count between WES and WGS was around 600 variants; this discrepancy is most likely explained by exome capture kit design and not mappability as we show below (Fig. 4). Surprisingly, we also found that some of the variant calling pipelines yield a very low number of pass-filter variants for both WES and WGS data (Supplementary Fig. 1b). The reasons for such behavior will be discussed in detail later.

### Systematic comparison of short read alignment and variant calling software

**Fig. 2:**
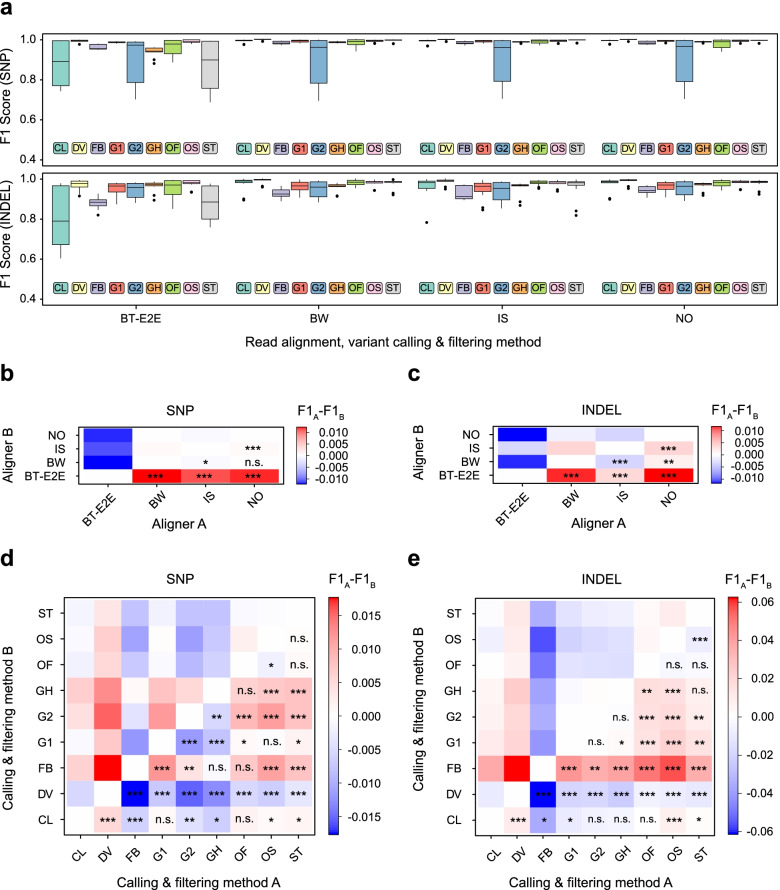
Statistical comparison of variant discovery pipelines’ performance. **A** Box plots representing the F1 scores for different combinations of aligners and variant callers. **B** - **E** Pairwise comparison of tool performance for read aligners (**B**, **C**) and variant callers (**D**, **E**) using pass-filter SNP (**B**, **D**) and indel (**C**, **E**) calls. On (**B**-**E**) the color of the cell corresponds to the median difference in F1 scores between the first tool (on the OX axis) and the second tool (on the OY axis); n.s. - the difference is not significant, * - *p* < 0.05, ** - *p* < 0.01, *** - *p* < 0.001 in the Wilcoxon paired signed rank test. Read aligners: BW - BWA MEM, BT-E2E - Bowtie2 (end-to-end mode), IS - isaac4, NO - Novoalign; variant callers and filtering strategies: CL - Clair3, DV - DeepVariant, G1 - GATK HaplotypeCaller with 1D CNN filtering, G2 - GATK HaplotypeCaller with 2D CNN filtering, GH - GATK HaplotypeCaller with recommended hard filters. ST - Strelka2, FB - Freebayes, OS - Octopus with standard filtering, OF - Octopus with random forest filtering

Usage of a set of 14 independent sequencing datasets from 7 individuals allows us not only to compare the performance of different tools, but also to assess the robustness and reproducibility of variant caller performance. To conduct such an analysis, we first examined the F1 scores (a harmonic mean of precision and recall) using variant calls inside CDS regions generated by each combination of read alignment and variant calling software (Fig. 2a). This analysis showed that variant callers seem to have a greater influence on the overall performance of a pipeline compared to short read aligners. Among all pipelines tested, a combination of BWA MEM with DeepVariant had the greatest F1 score, while DeepVariant showed best performance on both SNP and indels for any aligner. Among other solutions, the recently developed ones, including Strelka2, Clair3, and Octopus showed high accuracy when working with BWA, Isaac, or Novoalign and the default filters; at the same time, Clair3 and Strelka2 performance dramatically dropped when using Bowtie2 as the read aligner in both end-to-end and local modes (Fig. 2a, Supplementary Fig. 2). FreeBayes performed considerably worse than the aforementioned tools on both SNPs and indels, while GATK-HC had high accuracy only when 1D CNN or a hard filtering strategy was used. GATK-HC combined with the 2D CNN variant filtering showed the worst performance in SNP calling irrespective of the aligner used. Similarly, Octopus with the pretrained random forest filter also had high variance in the accuracy of SNP discovery, though the extent of such variability was much lower compared to GATK’s 2D CNN method. The reasons for this behavior of G2 and OF methods will be discussed in detail later.

We then sought to make a formal statistical comparison of read aligners and variant callers based on the benchmarking results. The structure of our dataset allows us to make such a comparison in a pairwise manner, and such comparison could in turn provide important information on the reproducibility of the differences between pipelines on various datasets. Pairwise comparison of all short read aligners showed that, despite low median differences in F1 scores (maximum value of F1 difference for a pair of aligners was 0.0038 for SNP and 0.0104 for indels), Isaac and Novoalign show the best performance on both SNPs (Fig. 2b) and indels (Fig. 2c); they are closely followed by BWA MEM. Bowtie2, on the other hand, performed considerably worse with both SNPs and indel variants (Fig. 2c). To our surprise, alignment with Bowtie2 in the local mode that allows soft clipping of read ends led to an even greater decrease in the performance of nearly all pipelines (Supplementary Fig. 2). Among all variant calling and filtering solutions, DeepVariant showed the best performance compared to all other tools (*p*-value < 0.001, Fig. 2d, e). Consistent with earlier observations, GATK-HC with the 2D CNN model performed reproducibly worse on SNPs than any other pipeline. At the same time, FreeBayes was the worst solution for indel discovery, with its F1 score being at least 5.7% lower compared to any other method (Fig. 2e). Clair3, Octopus, and Strelka2 performed almost equally well on SNPs and indels (*p*-value > 0.001) and were the closest runners-up to DeepVariant. Taken together, our statistical comparison demonstrated that the performance differences for variant callers and read aligners are reproducible when using different input data. The results also confirm that read alignment differences have a generally lower impact on the accuracy of variant discovery compared to variant calling software, with the exception of Bowtie2 which causes a substantial decrease in calling accuracy in both alignment modes tested.

We next questioned if the observed differences between variant calling pipelines can be mostly attributed to differences in precision or recall. To address this question, we compared precision and recall values reported for the same set of pipelines. This analysis revealed that the differences in precision were relatively small for all pipelines; at the same time, Clair3 showed markedly worse precision compared to other solutions while DeepVariant performed consistently better (*p* < 0.001). We also observed a surprising benefit in precision from otherwise underperforming Bowtie2 (*p* < 0.01, Supplementary Fig. S3). In contrast the difference in recall was much more substantial, with pipelines showing the lowest F1 scores showing the worst recall values (Supplementary Fig. S4). Given these observations, we conclude that the reproducible differences between variant calling software arise mostly from the power differences and not from different false positive rates.

**Fig. 3:**
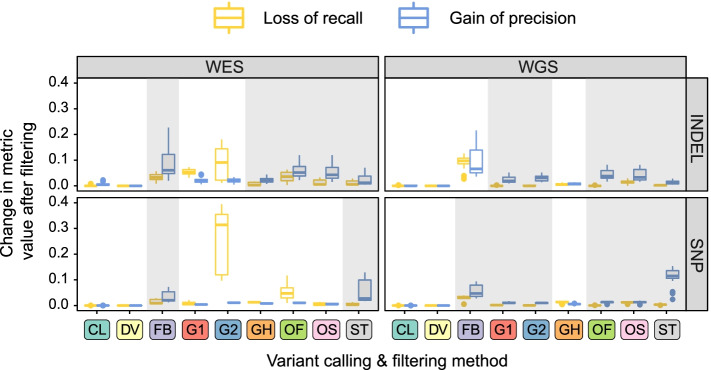
The necessity and possible benefit of variant filtering depends on the variant calling method. Shown are positive changes in precision (blue boxes) and negative changes in recall (yellow boxes) on SNPs and INDELs in WES and WGS data. Gray shading corresponds to cases where filtering is beneficial, i.e. gain in precision is greater than the loss of recall. Variant callers and filtering strategies: CL - Clair3, DV - DeepVariant, G1 - GATK HaplotypeCaller with 1D CNN filtering, G2 - GATK HaplotypeCaller with 2D CNN filtering, GH - GATK HaplotypeCaller with recommended hard filters. ST - Strelka2, FB - Freebayes, OS - Octopus with standard filtering, OF - Octopus with random forest filtering

Dramatic differences in recall values between variant calling pipelines prompted us to ask to what extent does variant filtering negatively influence the results. To test this, we first compared the F1 scores for the raw unfiltered data (Supplementary Fig. S5). Remarkably, we found that the differences between F1 scores of different pipelines on unfiltered data were less dramatic, though tools that showed best or worst performance on filtered data tended to have higher or lower F1 values on unfiltered data as well. This result indicates that variant filtering might decrease recall more than increase precision. To formally test this hypothesis, we compared the precision and recall values for variant sets before and after filtering with various filtering strategies. This analysis showed that the benefit from variant filtering heavily depends on the data type and variant calling method. For example, variant filtering was universally beneficial when using Strelka2 and FreeBayes; however, the effects of variant filtering in GATK and Octopus were different for WES and WGS samples (Fig. 3, Table 2). GATK’s neural network filters showed substantial gain in precision only for WGS data while having a significant negative impact on recall for WES samples. Filtering with either a standard filter or random forest model in Octopus was beneficial for WGS datasets but not for WES in which random forest filtering had a dramatic negative effect on recall. For DeepVariant, accurate comparison could not be made due to the lack of information about filtered genotypes.

**Table 2:** Effects of standard variant filtering methods on precision and recall

Caller and filtering strategy	Type	Filtering effects on SNPs^a^			Filtering effects on indels^a^		
		Raw calls F1	Precision gain	Recall loss	Raw calls F1	Precision gain	Recall loss
DeepVariant (default filter)	WGS	0.996	n.a.^b^	n.a.^b^	0.988	n.a.^b^	n.a.^b^
	WES	0.996	n.a^b^	n.a.^b^	0.990	n.a.^b^	n.a.^b^
Clair3 (default filter)	WGS	0.991	0.0	0.0	0.983	0.0	0.0
	WES	0.991	0.0	0.0	0.975	**0.0045**	0
Octopus (standard filter)	WGS	0.987	**0.0129**	−0.0120	0.973	**0.0328**	− 0.0153
	WES	0.992	**0.0049**	− 0.0028	0.967	**0.0429**	−0.0056
Octopus (random forest filter)	WGS	0.987	**0.0133**	−0.0003	0.973	**0.0379**	0.0
	WES	0.992	0.0104	**−0.0471**	0.967	**0.0518**	−0.0360
Strelka2 (default filter)	WGS	0.936	**0.1152**	− 0.0034	0.980	**0.0125**	−0.0026
	WES	0.980	**0.0274**	−0.0026	0.969	**0.0120**	−0.0056
GATK (1D CNN, tranches 99.9/99.5)	WGS	0.987	**0.0102**	−0.0014	0.971	**0.0189**	0.0
	WES	0.988	0.0039	**−0.0063**	0.962	0.0196	**−0.0532**
GATK (2D CNN, tranches 99.9/99.5)	WGS	0.987	**0.0099**	−0.0006	0.971	**0.0310**	0.0
	WES	0.988	0.0108	**−0.3548**	0.962	0.0219	**0.0909**
GATK (recommended hard filtering)	WGS	0.987	0.0056	**−0.0133**	0.971	**0.0067**	−0.0052
	WES	0.988	0.0078	**−0.0125**	0.962	**0.0217**	−0.0027
Freebayes (standard quality-based filter)	WGS	0.975	**0.0469**	−0.0301	0.950	0.0661	**−0.097**1
	WES	0.983	**0.0206**	−0.0087	0.948	**0.0695**	0.0328

^a^Median values across all samples are shown, greater absolute value (precision gain/recall loss) for each filtering strategy is highlighted in bold; ^b^effects of filtering on DeepVariant calls could not be assessed due to the structure of the output files

To sum up, we demonstrate that variant calling pipelines reproducibly differ in their performance of variant discovery (both for SNPs and indels). These differences are mostly driven by sensitivity of variant caller software, which could be substantially affected by variant filtering, especially for WES data. Pipelines based on DeepVariant consistently perform better than all other considered solutions, while usage of Bowtie2 and FreeBayes is not recommended due to large losses in accuracy, especially in certain combinations.

### Analysis of factors influencing the accuracy of variant discovery

We next went on to evaluate the influence of enrichment technology and other sequence-based factors on the accuracy of variant discovery with different tools. To do so, we conducted a stratified benchmarking of variant discovery pipelines using hap.py with both pre-defined stratifications provided by GIAB and custom region sets obtained from the coverage model [18].

**Fig. 4:**
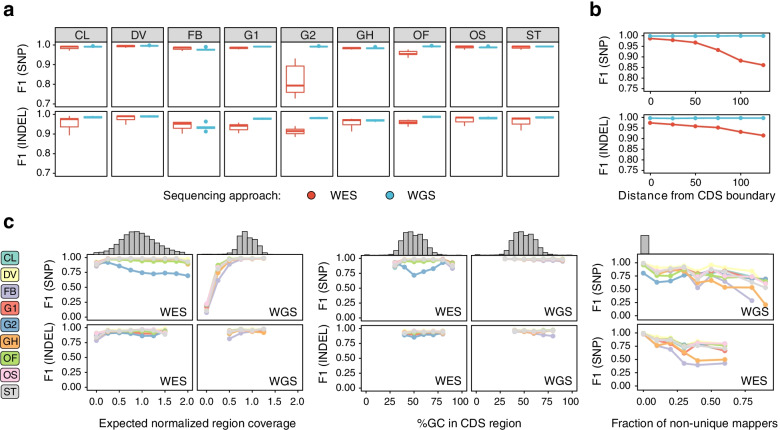
Dissection of factors affecting variant discovery pipelines’ performance. **a** Boxplots representing the F1 scores for SNP and indel calling with different variant callers in WES and WGS datasets. **b** The relationship between the distance from CDS boundary and the median F1 score of variant calling in WES and WGS data for SNP (top) and indel (bottom) variants. Results shown were obtained using the best-performing aligner (Novoalign) and three best-performing variant calling methods (DeepVariant, Strelka2, GATK-HC with hard filtering). **c** Comparison of the variant performance of variant callers in regions with different levels of expected normalized coverage (left), GC-content (middle), and fraction of non-unique mappers (right). Histograms on top of each plot represent the distribution of each parameter (coverage, GC content, MF) across GRCh37 CDS regions. Results shown on (**a**-**c**) were obtained using the best-performing read aligner (Novoalign v. 4.02.01). For other aligners, please see Supplementary Figs. S5, S6 and S7. Variant callers and filtering strategies: CL - Clair3, DV - DeepVariant, G1 - GATK HaplotypeCaller with 1D CNN filtering, G2 - GATK HaplotypeCaller with 2D CNN filtering, GH - GATK HaplotypeCaller with recommended hard filters. ST - Strelka2, OS and OF - Octopus with standard filtering and random forest filtering, respectively, FB - FreeBayes

We first compared the performance of different variant calling pipelines on WES and WGS data. Analysis of F1 scores for SNPs and indels revealed that, while accuracy of SNP discovery was comparable for WES and WGS, indel variants are harder to call using exome data (Fig. 4a, Supplementary Fig. S6). This result was reproducible across different read alignment and variant calling tools, with the exception of FreeBayes which showed comparably low efficiency of indel calling with both WES and WGS data as input, and DeepVariant which, to our surprise, had better median performance on WES compared to WGS data. Interestingly, GATK-HC with the 2D CNN variant scoring performed much worse on WES than on WGS data; in particular, due to very low accuracy of SNP filtration (we were unable to fix such behavior with parameter tuning). At the same time, scoring variants with the 2D CNN provided substantially higher accuracy for WGS data. A similar pattern can be seen, though to a much lower extent, for Octopus’ random forest filtering which also performs well on WGS but underperforms on WES data (Table 2, Table 3), particularly for SNPs. Hence, it can be concluded that the 2D scoring model in GATK and the random forest filter in Octopus should be applied only to whole-genome datasets with a more even coverage profile. Importantly, the aggregate differences between WES and WGS in SNP calling F1 scores for best variant calling pipelines were smaller compared to the differences in variant caller performance (median performance difference = 0.0003 for SNP and 0.006 for indels), suggesting that WES allows for a reasonably accurate variant discovery within CDS regions with the best performing variant calling solutions (Figs. 3, and 4).

**Table 3:** Aggregated median statistics of variant caller performance on WES and WGS data

Caller (filtering)^a^	Type	SNP F1	SNP Precision	SNP Recall	indel F1	indel Precision	indel Recall
DeepVariant	WGS	**0.995794**	**0.995365**	**0.996218**	**0.988316**	0.986772	0.992126
Octopus (standard)	WGS	0.987666	0.991172	0.984631	0.979687	0.985000	0.981771
Octopus (forest)	WGS	0.993052	0.990870	0.995244	0.987600	0.977995	**0.994595**
Strelka2	WGS	0.992320	0.991075	0.9929913	0.983985	0.984252	0.984375
Clair3	WGS	0.991248	0.987123	0.995530	0.984759	0.979798	0.989770
GATK (1D)	WGS	0.991736	0.988720	0.994891	0.977392	0.966921	0.992327
GATK (HF)	WGS	0.983078	0.983781	0.983338	0.969068	0.952618	0.984655
GATK (2D)	WGS	0.991804	0.988431	0.995803	0.981741	0.972010	0.991892
FreeBayes	WGS	0.976158	0.992710	0.960205	0.933873	**0.987988**	0.884910
DeepVariant	WES	**0.995837**	0.9972385	**0.994441**	**0.990379**	**0.989218**	**0.986523**
Octopus (standard)	WES	0.992911	0.992147	0.993656	0.983605	0.981818	0.980392
Octopus (forest)	WES	0.954045	**0.997630**	0.915830	0.959206	0.988796	0.931507
Strelka2	WES	0.992490	0.992002	0.992391	0.978279	0.975741	0.977961
Clair3	WES	0.991704	0.990249	0.991938	0.975506	0.970350	0.980716
GATK (1D)	WES	0.986426	0.984869	0.987764	0.942208	0.956044	0.922865
GATK (HF)	WES	0.985205	0.987470	0.983273	0.970658	0.958656	0.983193
GATK (2D)	WES	0.747232	0.991695	0.641138	0.914491	0.960000	0.900826
FreeBayes	WES	0.987447	0.991496	0.983301	0.952451	0.976667	0.931507

All values are given with respect to the Novoalign v.4.02.01 read alignment. Bold font corresponds to the best values for WGS and WES data. ^a^1D - 1D CNN model in GATK, 2D - 2D CNN model in GATK, HF - hard filtering with recommended parameters

Given our previous work on WES/WGS comparison [18], we hypothesized that lower WES performance in indel discovery was driven by regions in the vicinity of the CDS borders. To test this, we compared the median F1 score of variant discovery with different variant callers in regions located 25, 50, 75, 100, 125, or 150 bp away from the exon-intron boundary. This comparison revealed that the performance of best variant calling pipelines (Novoalign + DeepVariant/Strelka2/GATK-HC-1D) on SNPs declined modestly with increasing the distance from CDS for all pipelines (Fig. 4b, Supplementary Fig. S6), and the F1 metric value was comparable for CDS boundary and regions located up to 50 bp upstream and downstream of each CDS region. At the same time, reliability of indel variant discovery decreased more rapidly, and the overall accuracy of indel calling dropped significantly even at the distance of 25 bp from the CDS, and was lower than exome-wide F1 even at the exon boundary itself (Fig. 4b, Supplementary Fig. S7).

Remarkably, variant calling pipelines that performed worse in the general comparison (Fig. 2) also showed a greater rate of the accuracy decay while increasing the distance from the CDS boundary (Supplementary Fig. S7). We were surprised to discover that the trend was reversed for the GATK-HC with 2D CNN filtering and the random forest model in Octopus, which both showed higher accuracy in regions more distant from the CDS boundary. Given these observations, we can assume that high coverage might be one of the factors that negatively impacts the performance of these models. Taken together, our data suggest that variant calling in the regions flanking coding sequences (especially regions located no further than 50 bp away) is generally reliable for SNPs, but not indel variants when using WES data. Moreover, our observations suggest that the differences in indel calling accuracy between WES and WGS may be explained by low performance of variant callers near the exon boundaries. This offers an exciting opportunity to amend the probe design, greatly improving this specific aspect of WES performance and bringing it even closer to that of WGS.

We next turned to the analysis of other factors known to influence the reliability of variant calling. There are plenty of sources of coverage bias in both WES and WGS experiments, as detailed previously [18]. However, it is unclear which sources of reproducible coverage bias in WES and WGS impact the performance of variant calling software. To assess this, we compared the performance of variant discovery in regions with systematic differences in normalized coverage, GC content, and the fraction of non-uniquely mapped reads (multimapper fraction, MF) [18].

We started off by evaluating the dependence of the F1 score for each variant calling solution on the expected level of normalized sequence coverage as predicted by our recently proposed coverage model [18]. As expected, accuracy of all variant callers was decreased in regions with low normalized coverage in both WES and WGS. Surprisingly, we found that some variant calling pipelines also underperform in regions with high normalized coverage, especially for WES samples (Fig. 4c, left panel; Supplementary Fig. S8). Such reduced performance in high-coverage regions is the most pronounced for GATK neural network-based filtering methods; the most dramatic effect is seen for the 2D CNN method and the Octopus’ random forest filter, in line with earlier observations (Supplementary Fig. S8). Strelka2 is also slightly sensitive to high read depth, particularly for WGS datasets.

Yet another source of coverage bias in both WES and WGS is the GC-content of the sequence. We have previously shown that, while GC content is not a dominant determinant of poor sequencing coverage, extremely GC-rich or GC-poor regions tend to be substantially under-covered in WES. To assess the effects of the GC-content on the performance of variant calling software, we evaluated the F1 scores for each variant calling pipeline in regions with different GC content (split into 10% windows). We found that most variant callers’ performance drops significantly in extremely GC-rich regions (Fig. 4c, middle; Supplementary Fig. S8). Again, the effect of GC content on variant caller accuracy was the most pronounced for worst-performing variant calling methods such as FreeBayes; and the GC-content affects indel calling more than SNP calling in both WES and WGS. Importantly, variant calling in GC-rich regions was less efficient in WGS as well, although the relative drop in performance of variant callers in GC-rich regions is less significant for WGS than for WES (Fig. 4c).

At the same time, extremely GC-rich and GC-poor regions span not more than 80 kb (~ 0.2%) of the human coding sequence. Previously we demonstrated that mappability limitations of short reads play a much more important role in poor sequencing coverage in both WES and WGS. The performance of variant callers in completely repetitive coding regions (for example, in duplicated genes) cannot be evaluated as these regions are mostly unreachable for short reads. On the other hand, there are multiple CDS regions with imperfect repeats that are only partially covered by multimapping reads. To assess whether variant caller performance in such regions is decreased, we compared the F1 scores for all variant calling pipelines in regions with different proportions of non-uniquely mapped reads. This analysis showed that, indeed, accuracy of variant discovery is compromised in such regions (Fig. 4c, right panel). Similarly to previous comparisons, best-performing solutions such as DeepVariant tend to be less sensitive to read mappability issues, while conventional haplotype-based methods such as FreeBayes or GATK are the most sensitive to read mapping ambiguity. This observation suggests that machine learning-based software could at least partially overcome the mappability limitations and allow for variant discovery in regions with a high fraction of non-uniquely mapped reads.

Finally, we analyzed the total contribution of complex regions to the performance of variant calling and filtering methods. To this end, we compared the F1 scores obtained using GIAB v. 4.2 and v. 3.3 data (the high-confidence regions of the newer version include as much as 2 Mbp of hard-to-call regions). The comparison showed that the performance of all methods tested dropped when a broader set of regions was used (Supplementary Fig. S9). However, concordantly with all of the previous observations, the drop in performance was smaller for best-performing methods (such as DeepVariant) (median performance differences between GIAB versions < 0.005 for both SNPs and indels).

Taken together, our results demonstrate that, while coverage, GC-content, and mapping quality all affect accuracy of variant discovery in coding sequences, the best-performing variant calling pipelines are less sensitive to such confounding factors and perform better in all coding regions. At the same time, differences in the quality of variant discovery between WES and WGS are subtle and are mostly attributable to the low power of indel calling near the exon boundaries.

### Validation of variant caller performance using alternative datasets

Having dissected the major factors that affect variant caller performance on gold standard data, we next sought to assess whether the accuracy of variant calling will be similar on non-GIAB datasets. This analysis is especially important in the context of machine learning algorithms that might be prone to overfitting. This problem becomes increasingly relevant as NGS methods are increasingly applied to the analysis of poorly studied populations and ethnicities [19, 20]. To test for potential overturning of variant callers on GIAB data, we acquired an additional set of 3 WES and 3 WGS samples. The three exome samples came from our recent platform comparison study [18], while the three WGS samples were obtained from the NCBI SRA database and correspond to three individuals from the Yoruba population in Nigeria from 1000 Genomes project (NA18870, NA18871, NA18874) (Table 4, Supplementary Fig. S10). We decided to limit our analysis to BWA alignments and exclude GATK’s 2D CNN method as the one that is highly sensitive to coverage, as shown above (Fig. 4).

**Table 4:** Descriptive statistics of the additional non-GIAB samples used for overfitting analysis

Sample	Type	Source (SRA ID)	Ethnicity	Mean coverage	Fraction of 10x bases	Median variant count**
NA18870	WGS	ERX3266761	African	123.8	0.999	25,528
NA18871	WGS	ERX3266762	African	104.5	0.999	25,277
NA18874	WGS	ERX3270176	African	70.1	0.998	25,269
RUSZ02	WES	[18]	Russian	154.3	0.986	20,184
RUSZ05	WES	[18]	Russian	178.0	0.986	19,972
RUSZ07	WES	[18]	Russian	174.2	0.984	20,092

**Fig. 5:**
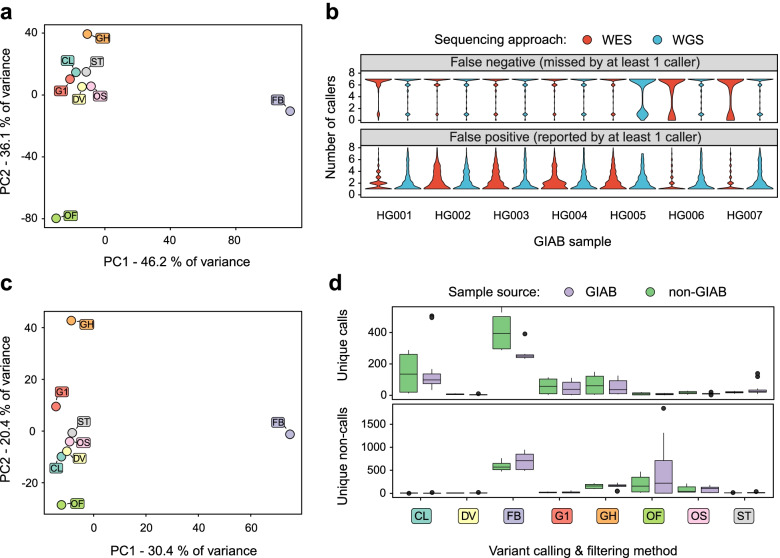
Variant calling and filtering methods perform similarly on GIAB and non-GIAB datasets. (**a**, **c**) A scatterplot showing the results of the principal component analysis in space of discordant variant calls on GIAB (**a**) and non-GIAB (**c**) data. **b** Distributions of the number of variant calling methods that detected each false negative (top) or false positive (bottom) variant. Note that the majority of FN and FP variants are represented by unique non-calls and unique calls, respectively. **d** Boxplots showing the number of unique calls (top) and unique non-calls (bottom) on GIAB and non-GIAB datasets for the indicated variant calling and filtering methods Variant callers and filtering strategies: CL - Clair3, DV - DeepVariant, G1 - GATK HaplotypeCaller with 1D CNN filtering, G2 - GATK HaplotypeCaller with 2D CNN filtering, GH - GATK HaplotypeCaller with recommended hard filters. ST - Strelka2, FB - Freebayes, OS - Octopus with standard filtering, OF - Octopus with random forest filtering

Evaluation of variant caller accuracy in the absence of gold standard variant calls is not straightforward and requires a certain metric that can be used as an indirect measure of false positive and false negative calls. We hypothesized that the concordance between different variant callers might be used as such an indirect measure. To test this assumption, we first selected all discordant variant calls from the main GIAB dataset, i.e. (a) false negative (FN) variants defined as true high-confidence variants missed by at least one of the variant callers; and (b) false positive variants defined as any false variant reported by at least one variant caller. We next applied principal component analysis to analyze the concordance between variant callers on this subset of FN and FP variants. PCA results showed that the concordance between variant callers is directly related to the performance, with the best-performing methods having high concordance and forming a dense cluster on a PCA biplot (Fig. 5a). In contrast, variant calling methods that were less efficient according to our evaluation (i.e., FreeBayes and GATJ-HC with hard filtering) or had issues with over-filtering of variants on WES samples, like Octopus’ random forest model, deviated significantly from the main group of points on the PCA, indicating lower concordance of variant calls.

We next asked whether the majority of discordant variant calls are represented by variants that were uniquely called or not called by only one variant calling pipeline. To answer this question, we plotted the distributions of the number of callers reporting each FN and FP variant in GIAB data. This analysis demonstrated that, indeed, most FN variants were missed by only one of the callers, while the majority of FP variants were reported by only one method (Fig. 5b). Taken together, the results of the concordance analysis on GIAB data showed that (i) the concordance between variant calling and filtering methods is indicative of their performance; and (ii) number of unique calls and unique non-calls can be used as an indirect measure of the accuracy of variant calling.

Given the aforementioned findings, we next analyzed the concordance of variant callers using a set of 6 non-GIAB samples described above. Principal component analysis of the discordant genotypes showed that the overall concordance of variant calling methods on non-GIAB samples is similar to GIAB, with FreeBayes, GATK-HC with hard filtering, and Octopus with random forest filtering showing substantial discordance of variant calls compared to the best-performing methods such as DeepVariant, Strelka2, and Octopus with standard filtering (Fig. 5c). At the same time, it’s worth noticing that the sum of variance explained by PC1 + PC2 is notably lower in case of non-GIAB samples (50.8% vs. 82.3%). In concordance with these findings, analysis of the number of unique calls and unique non-calls for each method revealed no substantial differences between GIAB and non-GIAB data for all variant callers except FreeBayes (Fig. 5d). In line with the rest of our results, this analysis showed that the total burden of unique calls (i.e., candidate FPs) and unique non-calls (i.e., candidate FNs) was the smallest for best-performing methods (DV, OS, and ST) and did not differ on GIAB and non-GIAB data.

Taken together, our analysis suggests that the performance of variant calling and filtering algorithms is similar on GIAB and non-GIAB data. This result implies that the results of the benchmarking presented above can be considered reliable and can be used as an unbiased measure of variant calling accuracy in coding regions of the human genome.

## Discussion

NGS methods have dramatically transformed the world of human genetics, both from the research and clinical perspective. WES and WGS are becoming the new standard in the diagnosis of Mendelian disease. However, despite the rapid spread and wide application of NGS-based methods in clinical practice, the average diagnostic rate is still below 50% for both trio-based WGS and trio-based WES [21]. Such low diagnostic rates are explained by a multitude of factors including inherent limitations of short-read sequencing technologies, imperfect human reference genome sequence, software limitations and, perhaps most importantly, incomplete understanding of the disease etiology and pathology [22]. In earlier publications, our and other groups have previously addressed some of the limitations associated with variability of WES/WGS coverage, short read mappability, and other related issues [4, 18, 23, 24]. In this study, we addressed the other major factor that plays an important role in variant discovery, namely, the performance of software pipelines for variant calling.

In contrast to other previously published studies, we undertook a more systematic approach by evaluating the performance of numerous different combinations of read aligners and variant callers using a large set of 14 WGS and WES samples. Such an approach allowed us to make estimates of the relative importance of different factors for accurate and reliable variant discovery. We show that variant calling software is the most important factor that greatly affects both SNP and indel calling. At the same time, the sequencing method (WGS or WES) has significant influence on the accuracy of indel detection, but virtually does not affect SNP calling (Table 3). Moreover, to our great surprise, we discovered that best-performing variant callers could show higher overall accuracy on WES than on WGS data. Finally, read alignment software has generally the lowest influence on the accuracy of variant discovery in coding sequences, although we would argue that the usage of Bowtie2 in human variant calling should be discouraged (Fig. 2b-c). According to our evaluations using public search engines, BWA is the most popular short-read aligner used in human variant calling; however, all of the profiled tools appear to be used to some extent. Taken together, these results highlight the fact that the correct choice of software tools for variant discovery is paramount for high-quality variant calling.

We showed that variant callers mostly differ in their sensitivity (Supplementary Figs. S3-S4) and the ability to accurately call variants in regions with poor sequencing coverage, extremely high or low GC-content, and/or non-zero fraction of multimappers (Fig. 4; Supplementary Fig. S8). We demonstrate that modern tools such as DeepVariant [10], Strelka2 [13], Clair3 [25], and Octopus [26], have the highest robustness and provide high accuracy of variant discovery for all tested datasets. These data support and expand previous observations that were made using individual gold standard samples [15, 16]. The best-performing solutions also tend to be less sensitive to these confounding factors such as depth of coverage and GC-content. Thus, our data suggest that recent developments in the field of variant calling software compensate for many of the limitations of short-read sequencing.

The DeepVariant method, the one that consistently shows the best accuracy of variant calling for both SNPs and indels, is based on a convolutional neural network model. Neural networks and other complex machine learning approaches are clearly the most promising for future development of bioinformatic software, including variant callers [27]. At the same time, in some cases (e.g., GATK CNNScoreVariants tool, or Octopus’ random forest variant filtering model) machine learning methods are more sensitive to artifacts and data quality, especially sequencing depth (Figs. 2, 3 and 4). This problem likely arises from overfitting, i.e. excessive tuning of the model to show best performance on the specific sets of training data. Such overtuning has also been demonstrated in the recent precisionFDA Truth 2 Challenge [28]. Unfortunately, machine learning models for variant calling and filtering have been trained using the same GIAB gold standard sequencing datasets that are usually used for benchmarking (including this study). This complicates the unbiased evaluation of the performance of these tools. To address this limitation and evaluate the performance of variant callers on other data, we employed a set of 6 non-GIAB samples (3 WES and 3 WGS datasets) from individuals of underrepresented ancestries (African and Russian). We showed that variant caller concordance can be used as a proxy to estimate the accuracy of variant calling (Fig. 5). Application of the concordance-based framework to the non-GIAB data showed that the variant callers are not substantially overtuned for GIAB data and show similar behavior in GIAB and non-GIAB datasets. Our results suggest that the machine learning methods currently used in variant calling are not sensitive to the individual’s ancestry or other properties of GIAB data; however, variation in sequencing depth and/or read distribution (for example, in WES data) may still greatly affect the results of variant calling with such methods (outside of DeepVariant).

Despite the lack of direct evidence of overfitting on GIAB samples we would still advocate the inclusion of a more diverse set of samples into the GIAB dataset, especially of African, Hispanic, or mixed ancestry. This would improve the models and increase robustness of the best-performing variant callers on different ethnical backgrounds, and increase the opportunity for cross-benchmarking. Recently, a new set of high-quality reference datasets have been generated for benchmarking of variant callers [29], and evaluation of several variant callers’ performance on these data corroborates the results present in our study. However, the new dataset is also based on the same individual GIAB genomes.

Given the results of our comparison, a simple set of recommendations could be made. All of the best-performing variant callers (DeepVariant, Strelka2, Clair3, or Octopus) could be used depending on the given tasks and resources. For example, DeepVariant and Clair3 were the slowest methods we tested, while Strelka2 was the fastest by a factor of 3 to 4. At the same time, we show that variant filtering is not always done in an optimal way, and that filtered variants should be retained and carefully examined for medical genetic applications. In particular, variant filtering with machine learning models (such as random forest available for Octopus or GATK’s CNN methods) is strongly not recommended on WES data unless the user has an in-house pretrained model designed specifically for such data type. It should also be noted that even the best variant callers could not make the correct call when the alignment is wrong. Thus, we would discourage the use of Bowtie2 which showed markedly lower performance in our benchmark in both end-to-end and local alignment modes. Finally, aside from notable outliers of HG006 and HG007, we confirm our previous conclusions that best WES solutions achieve performance that is very close to that of WGS, and should be considered a reliable and low-cost option for many applications. Some of the calling and filtering methods, however, are incompatible with WES data and should only be applied to WGS.

While we believe that our results provide important insights into the performance of state-of-the-art variant calling methods and highlight prospects for future development, several important issues have not been explicitly addressed in our work and may require further exploration. First, the version of the human reference genome assembly substantially influences the accuracy of variant discovery, beyond the problem of reference minor alleles reported previously [4]. Apart from major differences between commonly used GRCh37 (used in this study) and GRCh38 reference assemblies, inclusion of unplaced contigs, patches, and decoy sequences could influence both quantity and quality of variant calls. Additionally, a complete telomere-to-telomere (T2T) assembly of a human cell line has been published recently [30], representing the most dramatic change of the human reference genome in the last decade. Early evaluation of the T2T reference in variant calling has promised notable improvements in variant discovery [31], especially regarding the large and complex variants. However, the exact influence of using the complete reference on the performance of variant calling tools remains unknown.

Second, only high-confidence variant calling regions are routinely used for benchmarking of variant calling pipelines. While usage of such regions is useful to avoid the negative effects of coverage bias, it makes it harder to accurately compare variant caller sensitivity to low coverage, read mapping quality, and sequence complexity. Construction of a broader set of ground truth variant calls in non-high confidence regions would be useful for better benchmarking of variant calling methods, and for the development of robust new solutions for variant discovery in complex regions. The newer GIAB v. 4.2 truth set that was used in our study adds ~ 2 Mbp of difficult coding regions [28, 32], enabling a more nuanced comparison of pipeline performance. Side-by-side comparison of benchmarking results on GIAB v. 3.3 and GIAB v. 4.2 high-confidence regions shows that variant calling is less reliable in complex sequence regions (Supplementary Fig. S9). While the development of the GIAB v. 4.2 truth set represents a major step towards a comprehensive set of gold-standard variants for all coding regions of the genome, as much as 4.7 Mbp of human CDS sequence are either completely or partially not covered by the GIAB v. 4.2 high-confidence regions (Fig. 1c). This means further refinement of GIAB truth sets is indeed still required.

Third, we did not address the differences that could be introduced by the specific short-read sequencing technology, device, or library preparation method. Much of this variability would translate into systematic differences in coverage, which has been addressed before. On the other hand, differences in basecalling and error profiles could generate instrument-specific biases. However, a recent publication comparing NovaSeq 6000, HiSeq 4000, MGISEQ-2000, and BGISEQ-500 [14] has found the differences to be modest, at least among the Illumina machines.

Finally, software tools for bioinformatic analysis of NGS data are constantly improving. Besides accuracy, running time (which was not specifically evaluated in our analysis) may also present a serious problem when working with large genomic datasets. Multiple attempts have been made recently to achieve high scalability of the read alignment and variant calling software. These include, but are not limited to, development of a native Google Cloud Platform integration in the recent versions of GATK, faster reimplementation of the BWA MEM algorithm (BWA-MEM2, [33]), and many others. Constant development of novel methods and software tools suggests that large-scale stratified comparisons, like the one presented in our work, should be repeatedly conducted at least once in several years.

## Conclusions

The ongoing development of software for variant calling predicates the need for regular benchmarking of such tools. Our systematic comparison showed that variant caller typically influences the result more than read aligner, and that state-of-the-art variant callers, such as DeepVariant, Clair3, Octopus, and Strelka2 all allow for accurate variant discovery despite certain limitations. An indirect evaluation of pipeline performance using a set of GIAB and non-GIAB samples allowed us to conclude that variant callers do not show noticeable signs of overfitting for GIAB and perform comparably on non-GIAB samples, with DeepVariant showing the best performance on both datasets. Given such a robust performance of DeepVariant, we can argue that further development of gold standard datasets could further improve model training and push the accuracy of variant discovery closer to the limits of second-generation sequencing technologies.

## Methods

### Data acquisition

For our analysis, samples from seven GIAB individuals were selected: the NA12878 (HG001), three members of the Ashkenazi trio (HG002, HG003, and HG004), and three representatives of the Chinese trio (HG005, HG006, and HG007). Gold standard data for these samples were downloaded from the GIAB FTP repository (all WGS samples) or the NCBI Sequencing Read Archive SRA (all WES samples, respective SRA IDs: ERR1905890, SRR2962669, SRR2962692, SRR2962694, SRR2962693, SRR14724507, and SRR14724506). All sequencing datasets were generated using the Illumina Hiseq platform (HiSeq 2500 for all WGS datasets and HG002-HG005 WES samples; HiSeq 4000 for HG001 WES). Truth variant sets and high-confidence variant calling regions in BED format (release v. 3.3.2 or v. 4.2.1) were downloaded from the GIAB FTP data repository (https://ftp-trace.ncbi.nlm.nih.gov/giab/ftp/release/). Importantly, prior to being used in our analysis, the high-confidence regions for individual samples from GIAB were intersected using BEDtools, and only the regions found in all samples were retained. This totalled 30.4 Mbp of CDS sequences for v. 4.2.1 and 28.4 Mbp - for 3.3.2. Reference stratification BED files were retrieved from the GitHub repository provided by GIAB (https://github.com/genome-in-a-bottle/genome-stratifications/blob/master/GRCh37/v2.0-GRCh37-stratifications.tsv). Coding region intervals were extracted from the primary GENCODE v19 GTF annotation file. For the analysis of pathogenic variant distribution, pathogenic and likely pathogenic variants with non-conflicting interpretations were selected from ClinVar v.20211130.

For an additional comparison using non-GIAB datasets, a set of three WGS and three WES samples was selected. We have leveraged three exomes from the cohort used in our earlier analysis [18], and three genomes from the 1000 Genomes project’s YRI population (African ancestry) (SRA IDs ERX3266761, ERX3266762, ERX3270176).

### Variant calling pipelines

Variant discovery and filtering was performed using 45 different strategies, with 4 different read aligners (local and global modes were tested for Bowite2) and 9 modern variant callers. For read alignment, we used BWA MEM v.0.7.17 [34]; Bowtie2 v.2.3.5.1 [6], Novoalign v. 4.02.01 (http://novocraft.com/novoalign/) and Isaac v. 04.18.11.09 (https://github.com/Illumina/Isaac4). Novoalign was used under trial license obtained by R.A. and Y.A.B. Reads were aligned against the GRCh37.p13 primary human reference genome sequence. Aligned reads were pre-processed using GATK [9] v. 4.2.3 to mark duplicate read pairs. Coverage statistics were collected using GATK. GENCODE v19 exon coordinates were used to evaluate the depth and breadth of coverage. Coverage of the high-confidence CDS regions in all samples was analyzed using the results of read alignment with BWA MEM.

Variant calling was performed using six different tools and nine tool/filter combinations: FreeBayes v. 1.3.1 [35], GATK HaplotypeCaller (HC) v. 4.2.3 [8, 9], Strelka2 v. 2.9.10 [13], DeepVariant v. 1.2.0 [10], Clair3 v. 0.1-r8 [25], and Octopus v. 0.7.4 [26]. The DeepVariant caller was used with the default model for WGS or WES data, respectively. For Clair3, the default model for Illumina reads was used in all cases. For the GATK HaplotypeCaller, deduplicated reads in BAM format were also preprocessed using base quality score recalibration according to GATK Best Practices (https://gatk.broadinstitute.org/hc/en-us/articles/360035535932-Germline-short-variant-discovery-SNPs-Indels-). Variants were called in a single-sample mode, and the resulting VCF was subject to variant filtration using CNNScoreVariants with different model types (reference-based (1D) or reads-based (2D)) and hard filtering with the recommended parameters. For both CNN models, different tranche values were tested, and SNP tranche value of 99.9 and indel tranche value of 99.5 were used as showing the best performance. For CNN scoring, GATK v.4.2.0 was also used to assess the reproducibility of variant scoring results. For Strelka2, BAM files with marked duplicates were processed with Manta ([36]; https://github.com/Illumina/manta) to obtain a list of candidate indel sites. After Manta processing, Strelka2 v. 2.9.10 was configured using the default exome or genome mode and candidate calling regions obtained from Manta. Default filtering parameters were used. For FreeBayes we applied the default settings and filtered the resulting variant set by quality (QUAL < 30) and other recommended parameters using GATK. For Octopus, variants were first called in a default mode with standard filters applied. Next, a pretrained random forest model for germline variants was used to re-filter variants identified in the first step.

### Benchmarking of variant discovery tools

Benchmarking was performed using the hap.py tool, a reference implementation of the GA4GH recommendations for variant caller benchmarking [17]. RTGtools vcfeval was used as an engine for comparison [37]. For all samples and variant discovery pipelines, performance was evaluated using a set of GENCODE v19 exon regions with an additional 150 bp padding upstream and downstream of each exon. For WES samples, an additional BED file was provided to limit the analysis to targeted exon regions that are included in the design as indicated by the kit vendor. A common set of high-confidence variant calling intervals was used to make all comparisons as described above.

Reference stratification BED files were used for benchmarking alongside the custom BED. Several custom sets of regions were added to this set: (i) regions upstream and downstream of each CDS sequence (0–25 bp, 25–50 bp, 50–75 bp, 75–100 bp, 100–125 bp, and 125–150 bp), regions with varying fraction of reads with MQ = 0 (multimapper fraction, [18]), and regions with different expected normalized coverage obtained using a coverage model [18]

### Comparison of performance on GIAB and non-GIAB data

For comparison of the variant caller performance on GIAB and non-GIAB data, only BWA alignment results were used. For each sample, filtered variants obtained by each pipeline were transformed into a matrix form such that each row of the matrix represents a variant, and each column represents the variant calling method. In each cell of the matrix, the value indicated whether a given variant was reported (25) or not reported (0) by the specific variant caller. For GIAB samples, truth sets were additionally utilized to mark variants as true positive or false positive. All true variants that were missed by all variant callers for a given sample were added to the matrix and marked as false negatives. The resulting matrix contained 436,734 variant sites from each of the 20 samples used (14 GIAB and 6 non-GIAB ones).

For the analysis of the general variant caller concordance, principal component analysis was performed on a subset of the main matrix that did not contain true positive or false positive variants reported by all callers. PCA was performed separately on GIAB and non-GIAB data. For an indirect evaluation of the accuracy of variant calling, we calculated the number of unique calls and unique non-calls for each method. Unique calls were defined as variants that were reported by only one variant caller (such variants represent likely false positive calls), while unique non-calls were variants that were missed by a single variant caller (representing likely false negative calls).

### Statistical analysis

Statistical analysis of coverage statistics and benchmarking results was performed using R v. 4.1 with the following external packages: cowplot, colorRamps, ggplot2 [38], ggsci (https://github.com/nanxstats/ggsci), lattice, reshape2. Statistical comparison between short read alignment software and variant callers was performed using the paired Wilcoxon signed rank test.

## Supplementary Information

**Additional file 1:** Supplementary Figs. S1-S10.

**Additional file 2:** Supplementary Table S1.

## Data Availability

All data and code pertinent to the analysis presented here is available through GitHub: https://github.com/bioinf/caller_benchmark. GIAB samples were downloaded from the GIAB FTP repository (ftp://ftp-trace.ncbi.nlm.nih.gov/giab/ftp/release - all WGS samples) or the NCBI Sequencing Read Archive SRA (all WES samples, respective SRA IDs: ERR1905890, SRR2962669, SRR2962692, SRR2962694, SRR2962693, SRR14724507, and SRR14724506). Genomes of the three individuals of African ancestry (NA18870, NA18871, and NA18874) were also obtained from the SRA (SRA IDs: ERX3266761, ERX3266762, and ERX3270176).

## Abbreviations

WGS: Whole genome sequencing
WES: Whole exome sequencing
SNP: Single nucleotide polymorphism
CNN: Convolutional neural network
NGS: Next-generation sequencing
GIAB: Genome in a Bottle
NIST: National Institute of Standards
GA4GH: Global Alliance for Genomics and Health
