# Supplementary material for: Systematic benchmark of state-of-the-art variant calling pipelines identifies major factors affecting accuracy of coding sequence variant discovery
Source: BMC Genomics. 2022 Feb 22;23:155. doi: 10.1186/s12864-022-08365-3 (PMC8862519; doi:10.1186/s12864-022-08365-3)
Supplement: Supplementary file 1 — Additional file 1: Supplementary Figs. S1-S10. [file 12864_2022_8365_MOESM1_ESM.pdf]

Systematic benchmark of state-of-the-art variant calling pipelines identifies major factors affecting accuracy of coding sequence variant discovery

Yury A. Barbitoff[1,2,3,*], Ruslan Abasov[1,4], Varvara E. Tvorogova[1,3], Andrey S. Glotov[2] and Alexander V. Predeus[1]

[1]- Bioinformatics Institute, St. Petersburg, Russia
[2] - Dpt. of Genomic Medicine, D.O. Ott Research Institute of Obstetrics, Gynaecology and Reproductology, St. Petersburg, Russia
[3] - Dpt. of Genetics and Biotechnology, St. Petersburg State University, St. Petersburg, Russia
[4] - Dmitry Rogachev National Research Center of Pediatric Hematology-Oncology and Immunology, Moscow, Russia

Supplementary Files

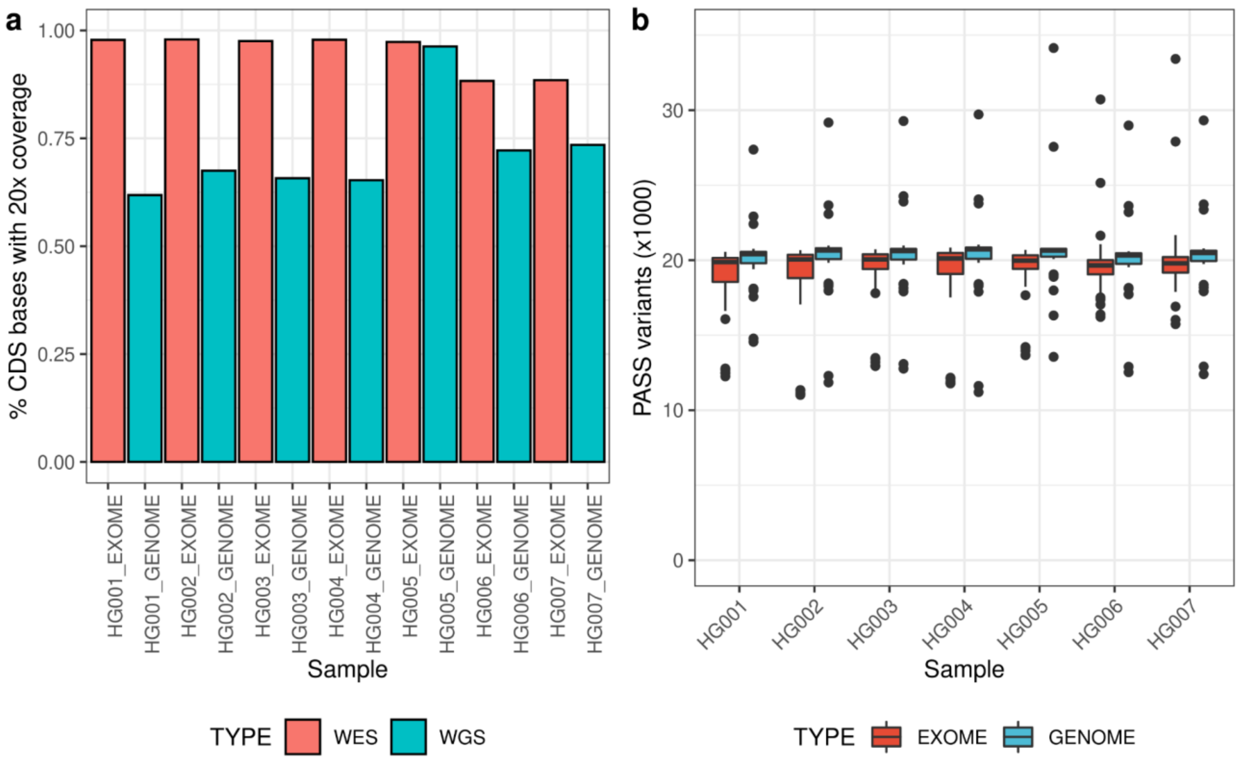

Figure S1. Additional descriptive statistics of the gold standard GIAB data used in the study. (a) A bar plot showing the fraction of GIAB v. 4.2 high-confidence CDS bases covered at least 20x in the indicated WES and WGS datasets. (b) Distribution of the number of PASS-filter variants identified by different pipelines in the indicated WES and WGS datasets.

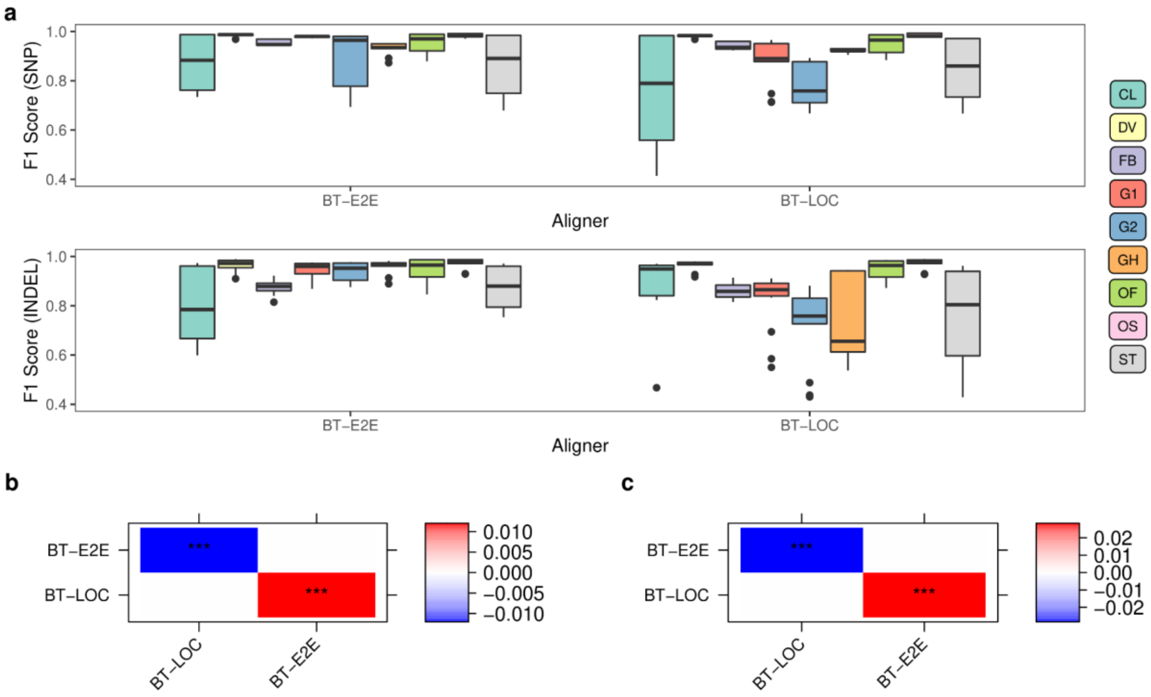

Figure S2. Enabling soft-clipping of read ends does not improve the Bowtie2 performance. (a) Box Plots representing the F1 scores for the indicated combinations of read alignment and variant calling methods. (b, c) A heatmap showing the median pairwise differences in F1 scores for SNPs (b) and indels (c) for pipelines based on different modes of Bowtie2 alignment. BT-LOC - Bowtie2 in local mode (soft clipping enabled); BT-E2E - Bowtie2 in end-to-end mode (soft clipping disabled). Variant callers and filtering strategies: CL - Clair3, DV - DeepVariant, G1 - GATK HaplotypeCaller with 1D CNN filtering, G2 - GATK HaplotypeCaller with 2D CNN filtering, GH - GATK HaplotypeCaller with recommended hard filters. ST - Strelka2, FB - Freebayes, OS - Octopus with standard filtering, OF - Octopus with random forest filtering.

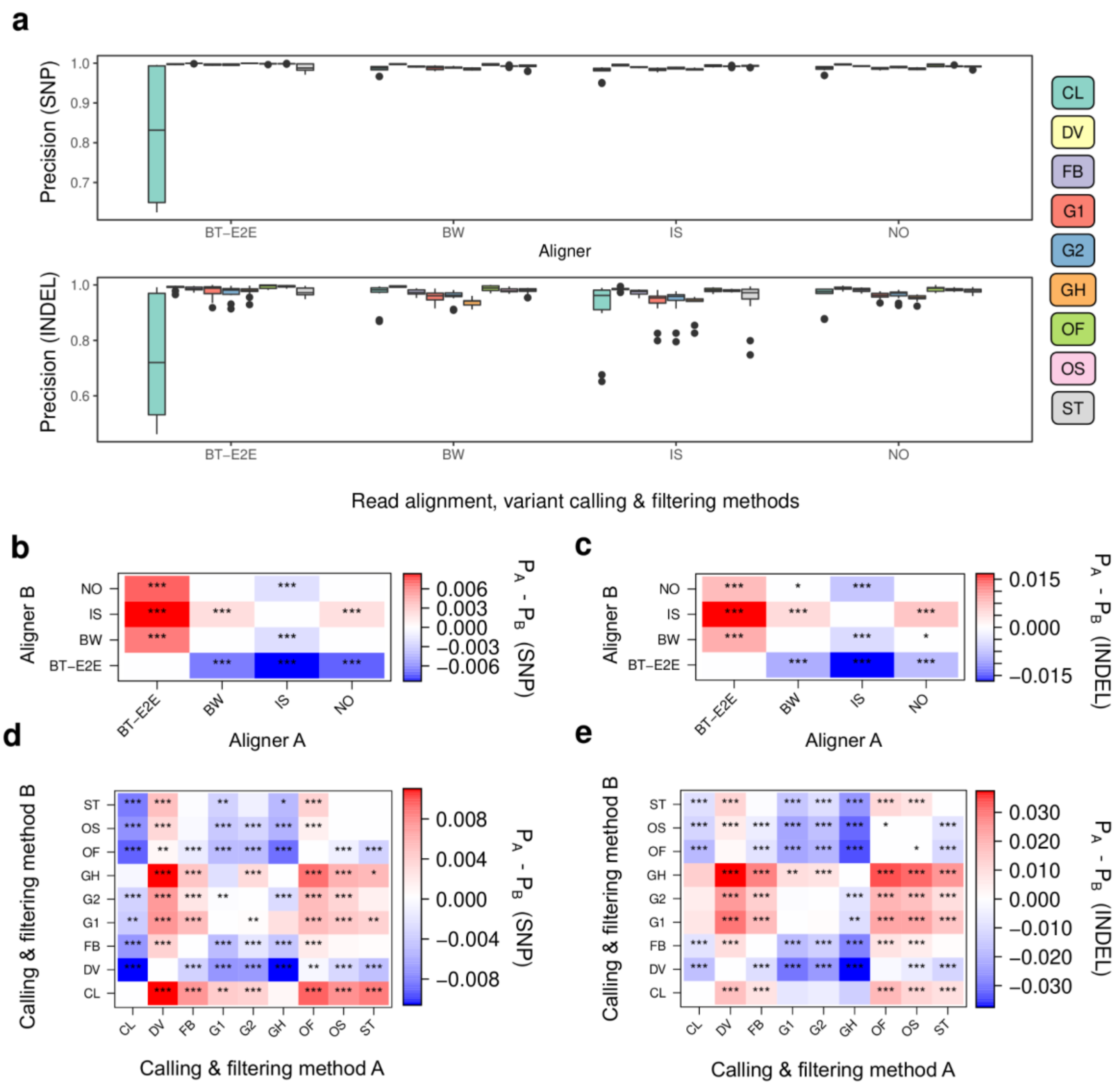

Figure S3. Comparison of the precision of alignment and variant calling methods. Top, box plots representing precision values for SNP and indel calling for all combinations of read alignment and variant calling software. Bottom, matched pairwise comparison of read alignment or variant callers in terms of precision of SNP and indel discovery. Read aligners: BW - BWA MEM, BT-E2E - Bowtie2 (end-to-end mode), IS - isaac4, NO - Novoalign; variant callers and filtering strategies: CL - Clair3, DV - DeepVariant, G1 - GATK HaplotypeCaller with 1D CNN filtering, G2 - GATK HaplotypeCaller with 2D CNN filtering, GH - GATK HaplotypeCaller with recommended hard filters. ST - Strelka2, FB - Freebayes, OS - Octopus with standard filtering, OF - Octopus with random forest filtering.

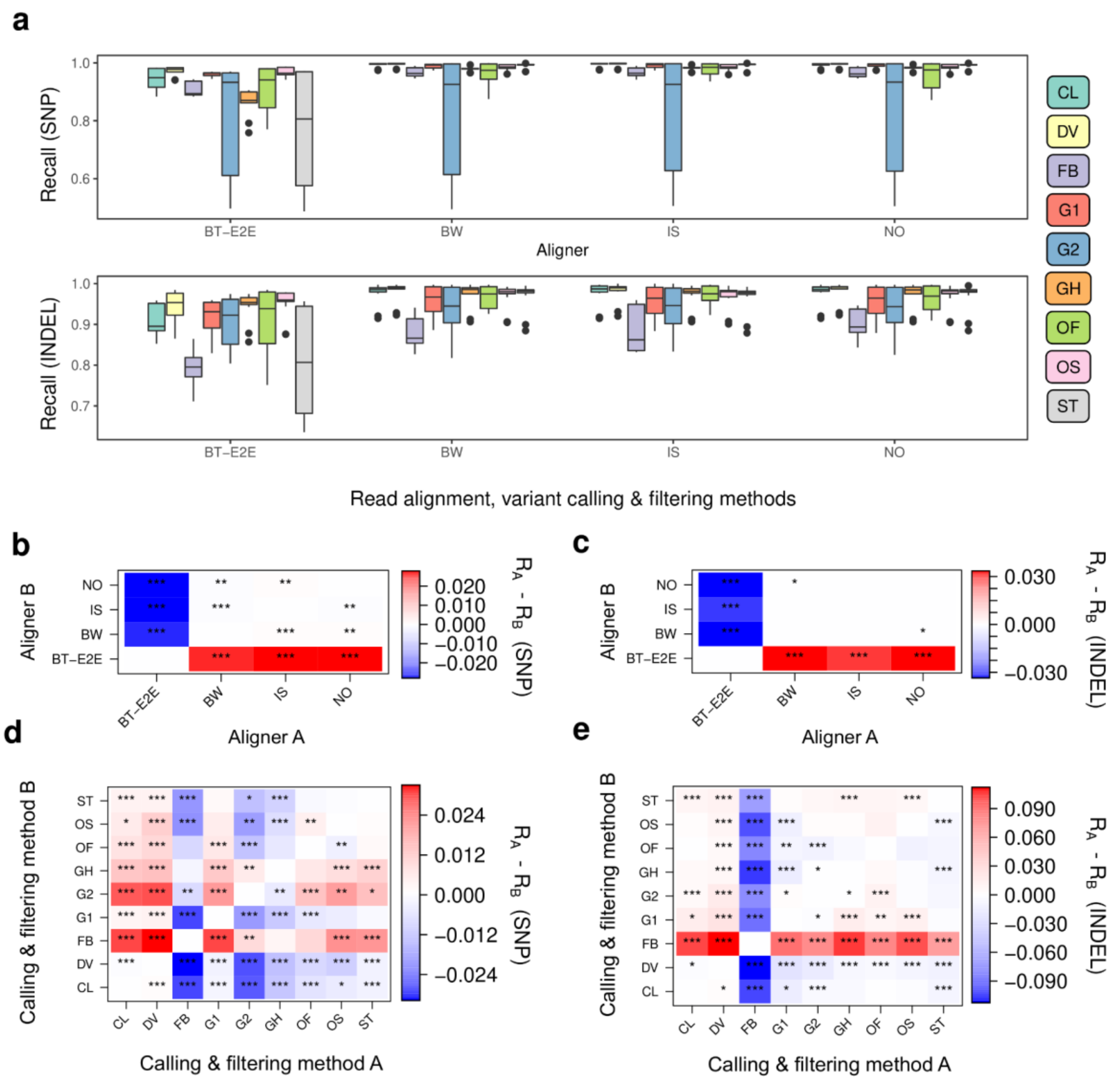

Figure S4. Comparison of the recall of alignment and variant calling methods. Top, box plots representing precision values for SNP and indel calling for all combinations of read alignment and variant calling software. Bottom, matched pairwise comparison of read alignment or variant callers in terms of precision of SNP and indel discovery. Read aligners: BW - BWA MEM, BT - Bowtie2, IS - isaac4, NO - Novoalign; variant callers and filtering strategies: CL - Clair3, DV - DeepVariant, G1 - GATK HaplotypeCaller with 1D CNN filtering, G2 - GATK HaplotypeCaller with 2D CNN filtering, GH - GATK HaplotypeCaller with recommended hard filters. ST - Strelka2, FB - Freebayes, OS - Octopus with standard filtering, OF - Octopus with random forest filtering.

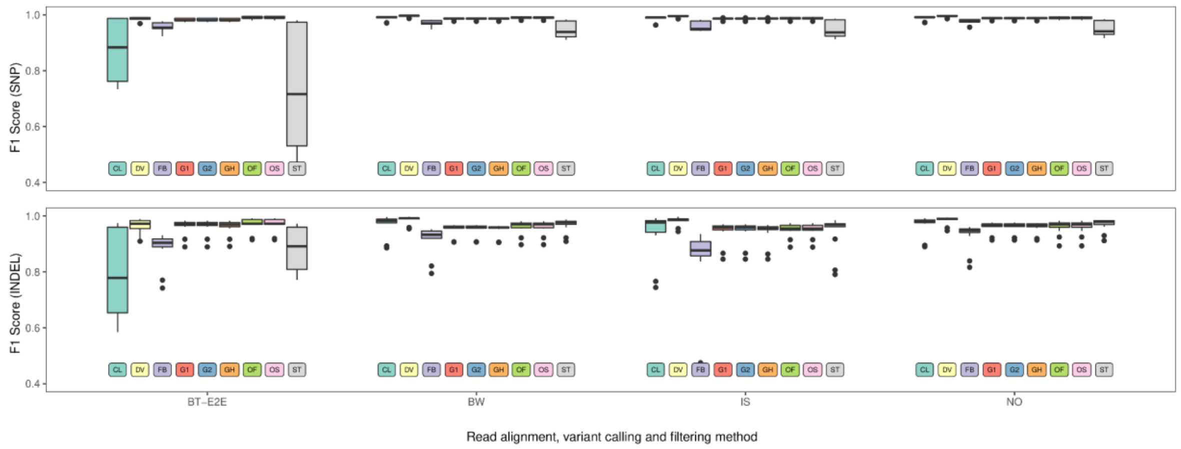

Figure S5. Comparison of the F1 scores of variant calling pipeline prior to applying filters. Please note that all pipelines based on GATK have identical F1 scores before filtering. Read aligners: BW - BWA MEM, BT-E2E - Bowtie2 (end-to-end mode), IS - isaac4, NO - Novoalign; variant callers and filtering strategies: CL - Clair3, DV - DeepVariant, G1 - GATK HaplotypeCaller with 1D CNN filtering, G2 - GATK HaplotypeCaller with 2D CNN filtering, GH - GATK HaplotypeCaller with recommended hard filters. ST - Strelka2, FB - Freebayes, OS - Octopus with standard filtering, OF - Octopus with random forest filtering.

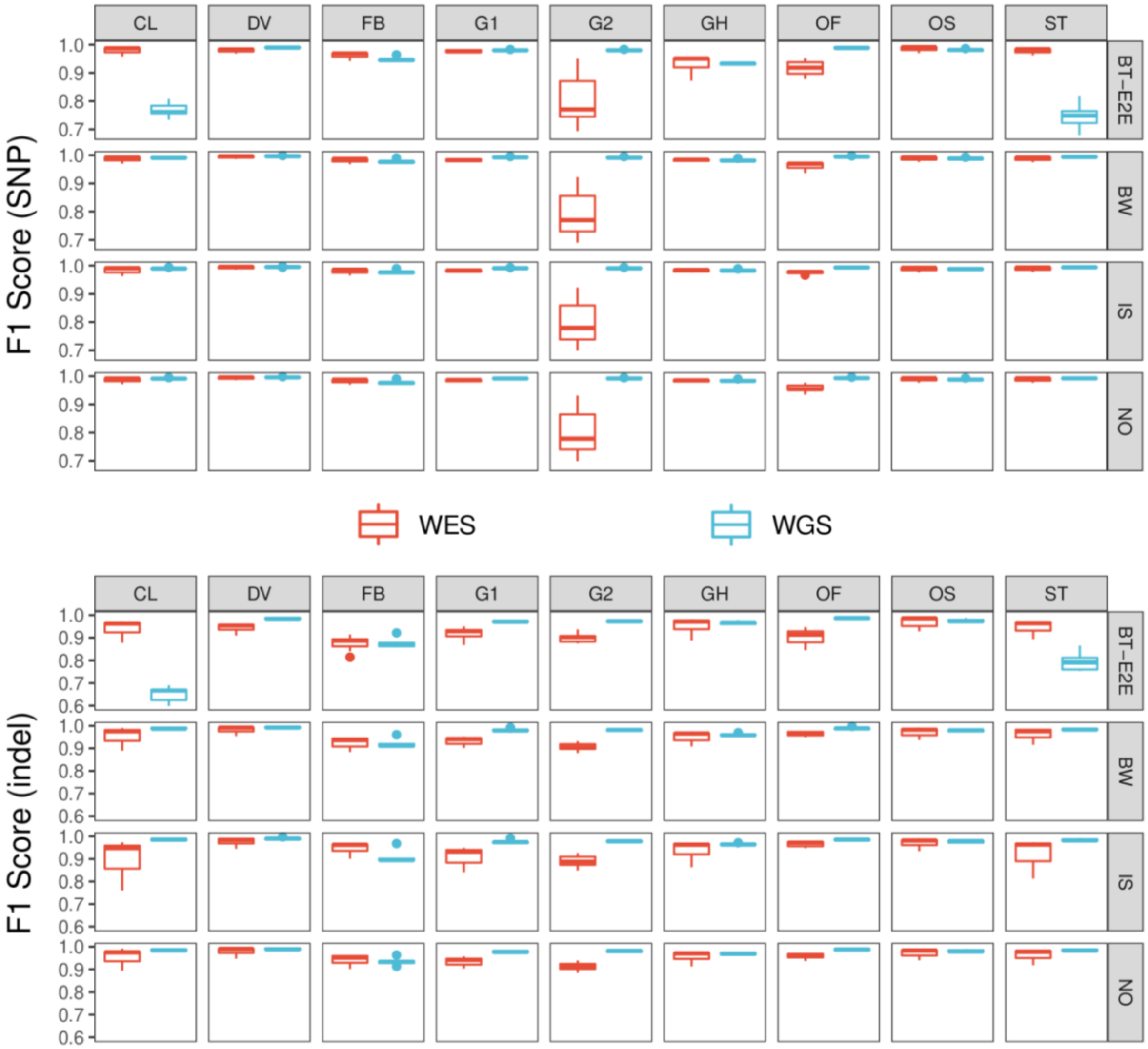

Figure S6. Comparison of variant calling pipelines' performance on WES and WGS data. F1 scores for SNP (top) or indel (bottom) calling are shown. Read aligners: BW - BWA MEM, BT-E2E - Bowtie2 (end-to-end mode), IS - isaac4, NO - Novoalign; variant callers and filtering strategies: CL - Clair3, DV - DeepVariant, G1 - GATK HaplotypeCaller with 1D CNN filtering, G2 - GATK HaplotypeCaller with 2D CNN filtering, GH - GATK HaplotypeCaller with recommended hard filters. ST - Strelka2, FB - Freebayes, OS - Octopus with standard filtering, OF - Octopus with random forest filtering.

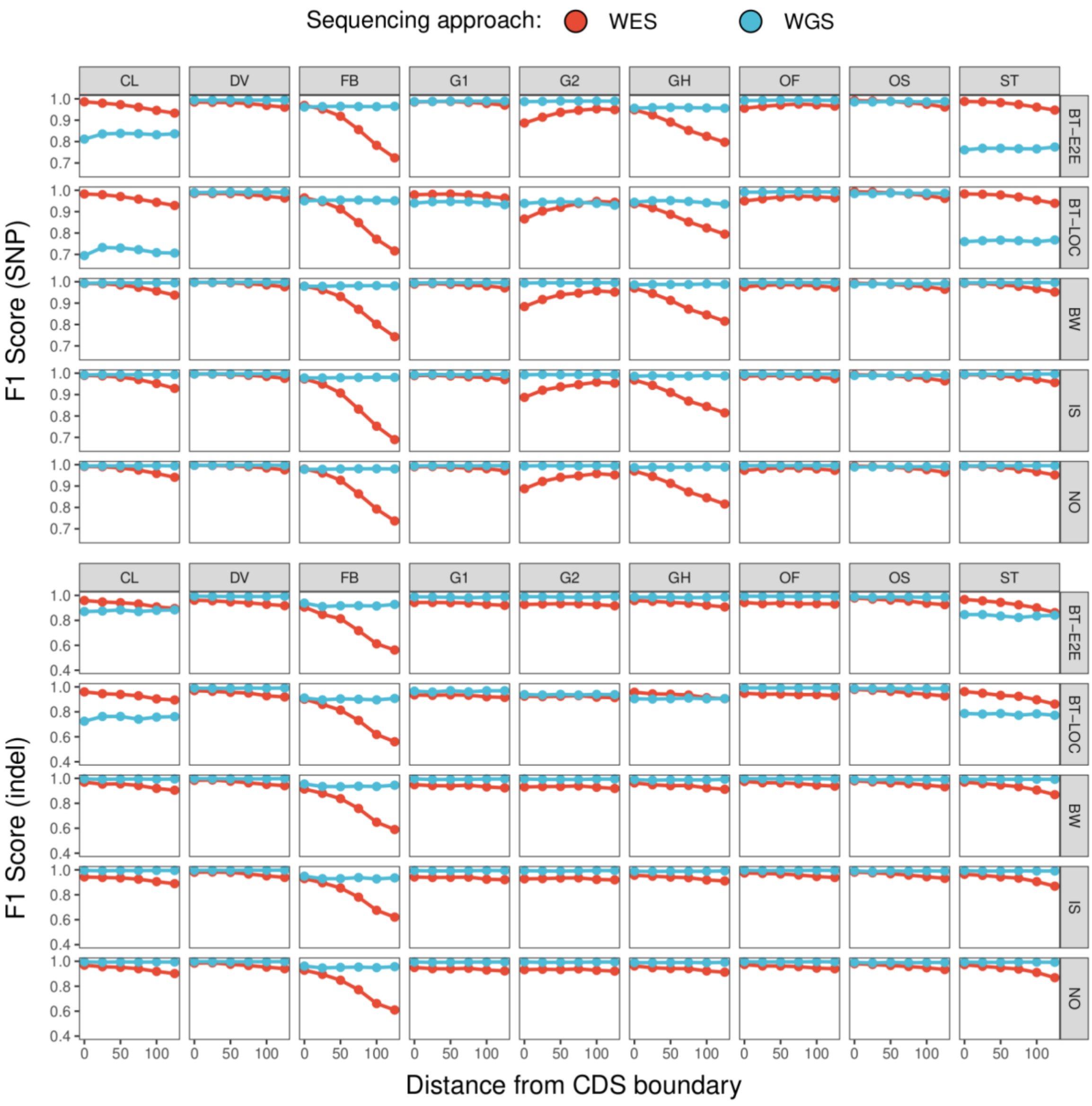

Figure S7. Accuracy of variant discovery in the vicinity of CDS regions for all variant calling pipelines. F1 scores for SNP (top) and indel (bottom) calling are shown. Read aligners: BW - BWA MEM, BT-E2E - Bowtie2 (end-to-end mode), BT-LOC - Bowtie2 (local mode). IS - isaac4, NO - Novoalign; variant callers and filtering strategies: CL - Clair3, DV - DeepVariant, G1 - GATK HaplotypeCaller with 1D CNN filtering, G2 - GATK HaplotypeCaller with 2D CNN filtering, GH - GATK HaplotypeCaller with recommended hard filters. ST - Strelka2, FB - Freebayes, OS - Octopus with standard filtering, OF - Octopus with random forest filtering.

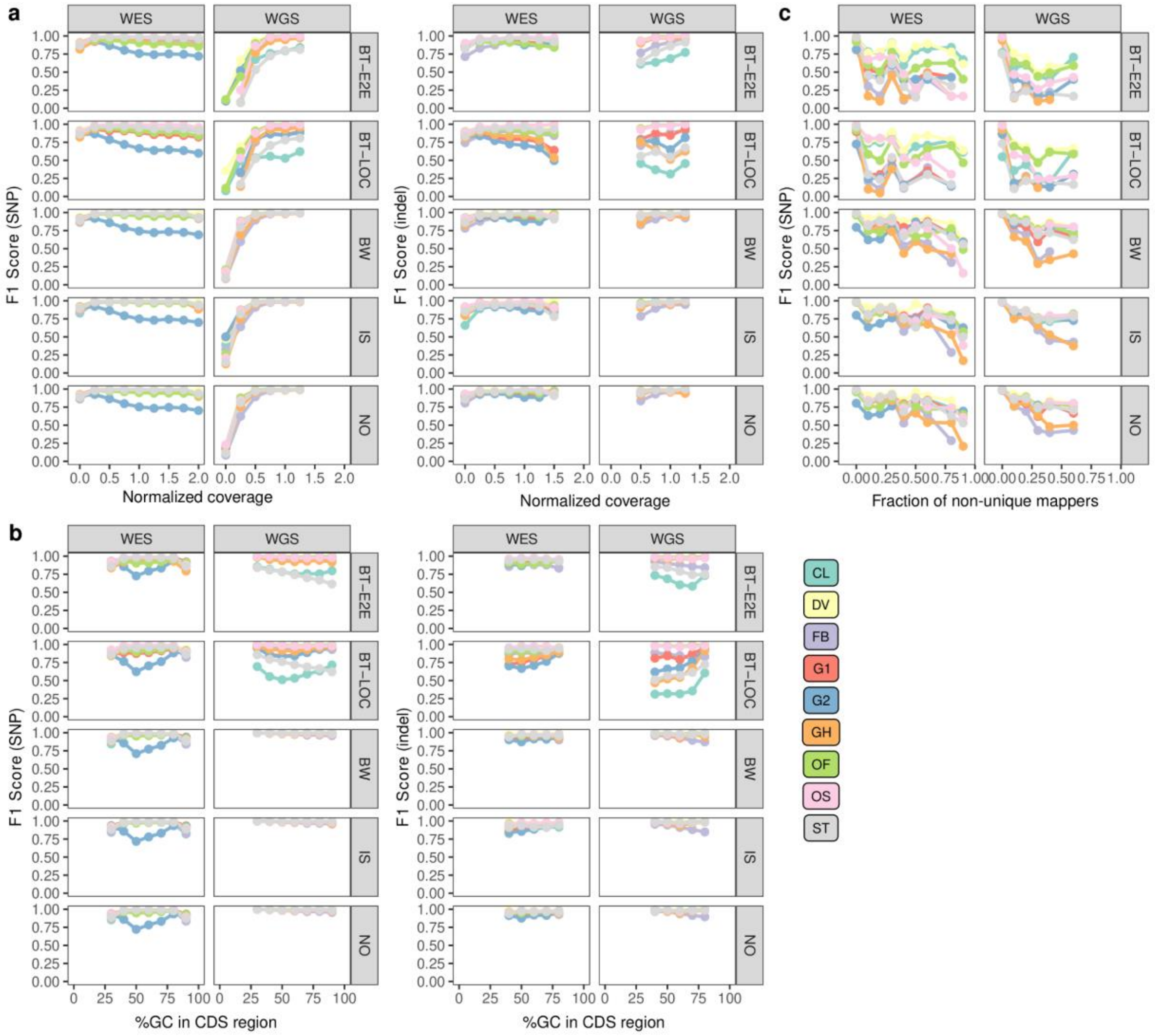

Figure S8. Comparison of the variant calling pipelines' performance in regions with different levels of expected normalized coverage (a), GC-content (b), and fraction of non-unique mappers (c). Histograms on top of each plot represent the distribution of each parameter (coverage, GC content, MF) across GRCh37 CDS regions. Read aligners: BW - BWA MEM, BT-E2E - Bowtie2 (end-to-end mode), BT-LOC - Bowtie2 (local mode). IS - isaac4, NO - Novoalign; variant callers and filtering strategies: CL - Clair3, DV - DeepVariant, G1 - GATK HaplotypeCaller with 1D CNN filtering, G2 - GATK HaplotypeCaller with 2D CNN filtering, GH - GATK HaplotypeCaller with recommended hard filters. ST - Strelka2, FB - Freebayes, OS - Octopus with standard filtering, OF - Octopus with random forest filtering.

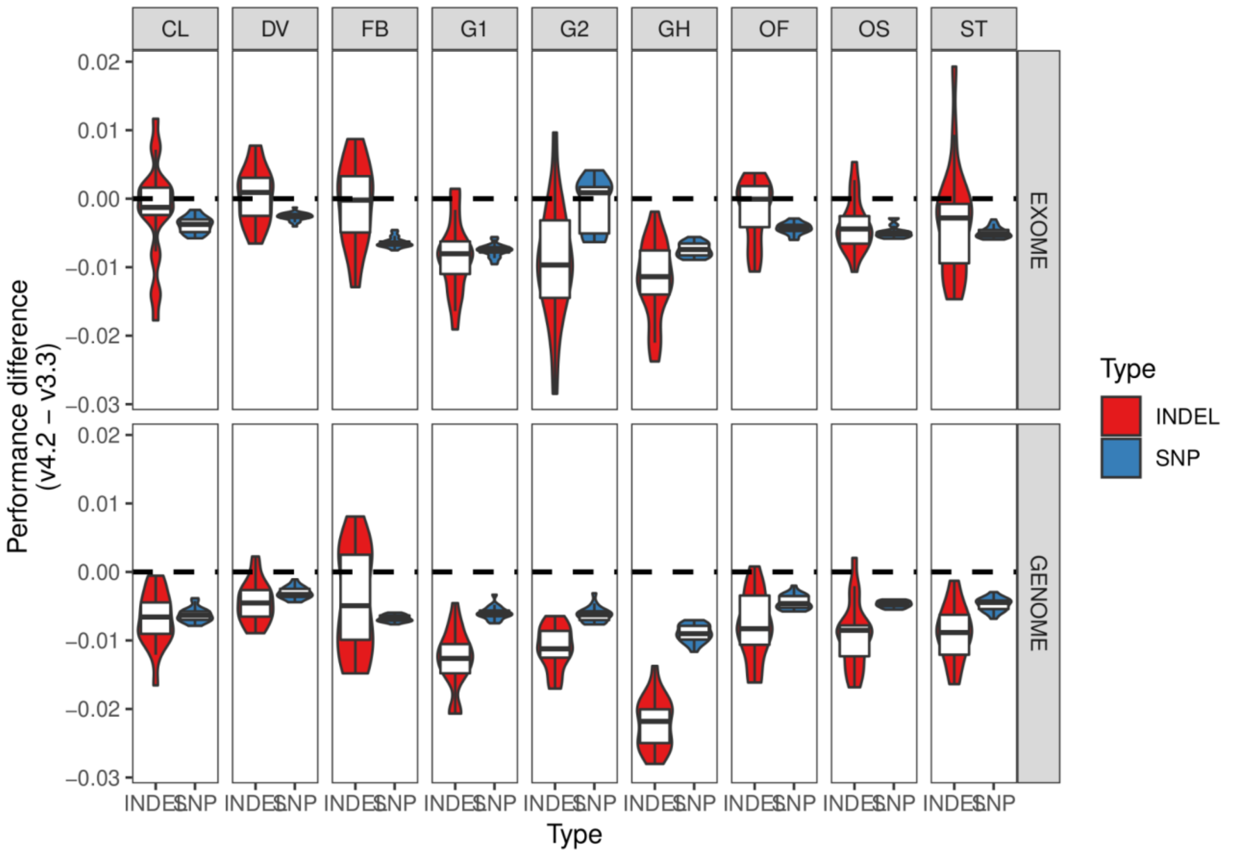

Figure S9. Almost all variant callers perform worse on GIAB v. 4.2 data compared to GIAB v. 3.3. Shown are the distributions of pairwise differences in F1 scores obtained when benchmarking variant callers against GIAB v. 4.2 or v. 3.3 for SNPs (blue) or indels (red) on WES and WGS data. Variant callers and filtering strategies: CL - Clair3, DV - DeepVariant, G1 - GATK HaplotypeCaller with 1D CNN filtering, G2 - GATK HaplotypeCaller with 2D CNN filtering, GH - GATK HaplotypeCaller with recommended hard filters. ST - Strelka2, FB - Freebayes, OS - Octopus with standard filtering, OF - Octopus with random forest filtering.

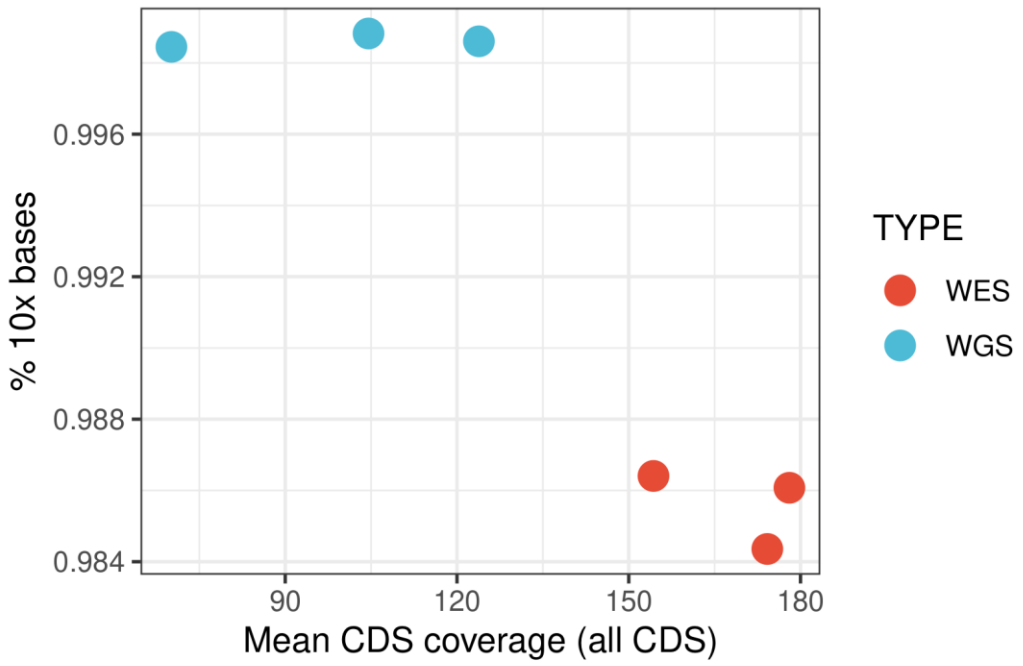

Figure S10. Sequencing quality statistics for the additional non-GIAB datasets. Shown is the scatterplot of mean coverage of GIAB v. 4.2 high-confidence regions and the fraction of CDS bases covered at least 10x.

Supplementary Tables

Table S1. Precision, recall, and F1 statistics of each variant caller for each sample, data type, and variant type (SNP/indel). Table is available as a Supplementary File.
